# Supplementary material for: Psychological characteristics associated with COVID-19 vaccine hesitancy and resistance in Ireland and the United Kingdom
Source: Nat Commun. 2021 Jan 4;12:29. doi: 10.1038/s41467-020-20226-9 (PMC7782692; doi:10.1038/s41467-020-20226-9)
Supplement: Supplementary file 2 — Reporting Summary [file 41467_2020_20226_MOESM2_ESM.pdf]

nature research

Corresponding author(s): Jamie Murphy

Last updated by author(s): Nov 6, 2020

Reporting Summary

Nature Research wishes to improve the reproducibility of the work that we publish. This form provides structure for consistency and transparency in reporting. For further information on Nature Research policies, see our Editorial Policies and the Editorial Policy Checklist.

Statistics

For all statistical analyses, confirm that the following items are present in the figure legend, table legend, main text, or Methods section.

n/a	Confirmed	
☐	☒	The exact sample size (*n*) for each experimental group/condition, given as a discrete number and unit of measurement
☐	☒	A statement on whether measurements were taken from distinct samples or whether the same sample was measured repeatedly
☐	☐	The statistical test(s) used AND whether they are one- or two-sided<br>*Only common tests should be described solely by name; describe more complex techniques in the Methods section.*
☐	☒	A description of all covariates tested
☐	☒	A description of any assumptions or corrections, such as tests of normality and adjustment for multiple comparisons
☐	☒	A full description of the statistical parameters including central tendency (e.g. means) or other basic estimates (e.g. regression coefficient) AND variation (e.g. standard deviation) or associated estimates of uncertainty (e.g. confidence intervals)
☐	☒	For null hypothesis testing, the test statistic (e.g. *F*, *t*, *r*) with confidence intervals, effect sizes, degrees of freedom and *P* value noted<br>*Give P values as exact values whenever suitable.*
☒	☐	For Bayesian analysis, information on the choice of priors and Markov chain Monte Carlo settings
☒	☐	For hierarchical and complex designs, identification of the appropriate level for tests and full reporting of outcomes
☐	☒	Estimates of effect sizes (e.g. Cohen's *d*, Pearson's *r*), indicating how they were calculated

Our web collection on statistics for biologists contains articles on many of the points above.

Software and code

Policy information about availability of computer code

Data collection	Fieldwork and data collection was conducted by the survey company Qualtrics, which has completed more than 15,000 projects across 2,500 universities worldwide. The data was generated using Qualtrics software, Version - March 2020.
Data analysis	All data analyses were conducted using: IBM Corp. Released 2017. IBM SPSS Statistics for Windows, Version 25.0. Armonk, NY: IBM Corp.

For manuscripts utilizing custom algorithms or software that are central to the research but not yet described in published literature, software must be made available to editors and reviewers. We strongly encourage code deposition in a community repository (e.g. GitHub). See the Nature Research guidelines for submitting code & software for further information.

Data

Policy information about availability of data

All manuscripts must include a data availability statement. This statement should provide the following information, where applicable:

- Accession codes, unique identifiers, or web links for publicly available datasets
- A list of figures that have associated raw data
- A description of any restrictions on data availability

The datasets generated and/or analysed during the current study are available in the Open Science Framework (OSF) repository, and can be accessed here: https://osf.io/58swj/. Source data are also provided with this paper.

Field-specific reporting

Please select the one below that is the best fit for your research. If you are not sure, read the appropriate sections before making your selection.

☐ Life sciences ☒ Behavioural & social sciences ☐ Ecological, evolutionary & environmental sciences

For a reference copy of the document with all sections, see nature.com/documents/nr-reporting-summary-flat.pdf

Behavioural & social sciences study design

All studies must disclose on these points even when the disclosure is negative.

Study description	A study identifying the socio-demographic and psychological correlates of COVID-19 vaccine acceptance, hesitancy and resistance in Ireland and the UK
Research sample	Data was collected from nationally representative samples of the general adult populations of Ireland (N = 1,041) and the UK (N = 2,025). These data were collected as part of the COVID-19 Psychological Research Consortium (C19PRC) Study to track the mental health and societal impact of the pandemic across both countries. The Irish sample: was 51.5% female, had a mean age of 44.97 years (SD=15.76), comprised 70.7% Irish born respondents, comprised 25.2% respondents of ethnic minority status, 28.8% rural dwellers, comprised 1.2% respondents without an educational qualification, comprised 24.6% respondents with an income between €0 and €20,000 and 21.9% respondents with an income above €50,000, comprised 59% respondents in part or full time employment, comprised 69.8% respondents who identified as Christian, comprised 18.4% respondents who were the only adult in the household, and 39.7% respondents who lived with at least one child. The UK sample: was 51.7% female, had a mean age of 45.45 years (SD=15.90), comprised 90.6% British born respondents, comprised 14.5% respondents of ethnic minority status, 16.5% rural dwellers, comprised 2.9% respondents without an educational qualification, comprised 20.2% respondents with an income between £0 and £15,490 and 20.2% respondents with an income above £57,931, comprised 63.8% respondents in part or full time employment, comprised 50.4% respondents who identified as Christian, comprised 22.4% respondents who were the only adult in the household, and 29.2% respondents who lived with at least one child.
Sampling strategy	Stratified quota sampling was used to ensure that the sample characteristics of sex, age, and geographical distribution matched known population parameters for the Irish population while age, sex and income matched known population parameters for the UK population. The Qualtrics survey company (see Data collection section below) proceeded as follows: (1) respondents in 'hard to reach' quota groups (e.g. young adults in the highest income bands) were prioritised and targeted first; (2) next, the focus shifted to allow the quotas to 'fill up' naturally, without specific targeting; and (3) finally, a switch back to targeting respondents to fill incomplete quotas ensued. Power analyses were conducted to determine the optimal sample sizes for both countries. As the C19PRC Study was primarily concerned with tracking mental health disorders (depression, generalized anxiety disorder [GAD], and posttraumatic stress disorder [PTSD]) in the general population, sample size calculations were based on existing prevalence estimates for these disorders. In Ireland and the UK, the estimated prevalence of PTSD is 5% and 4%, respectively, and lower than the prevalence estimates of depression and generalized anxiety. To detect a disorder with a prevalence of 4%, with precision of 1%, and 95% confidence level, a sample size of 1476 was required. The survey company used to collect the data could only guarantee a maximum sample size of 1,000 participants in Ireland, whereas a larger sample could be obtained in the UK. This is a consequence of the much smaller population of Ireland (4.9 million people) compared to the UK (66.7 million people). Therefore, the target sample size in Ireland was set at 1,000 which, holding all other parameters in the sample size calculation equal, resulted in a precision of 1.21%. In the UK, a target sample was set at 2,000 to increases the number of 'cases' detected because of the intention to track changes in the mental health problems in the population over time.
Data collection	Fieldwork and data collection was conducted by the survey company Qualtrics. As an aggregator of panels, Qualtrics provides the online platform to securely house data and leverages partners to connect with respondents. Qualtrics recruits study participants from traditional, actively managed, double-opt-in market research panels, which are used for corporate and academic market research only. All of Qualtrics' partners are members of the European Society for Opinion and Marketing Research (ESOMAR), the Council of American Survey Research Organisations (CASRO) and other national organizations. The 'opt-in for market research' process requires respondents to submit an initial registration form requesting to participate in market research studies. Potential respondents build their profile from a standardized list of questions. Potential respondents could have been alerted to the C19PRC study by Qualtrics in one of two ways: (1) they opted to enter studies they are eligible for themselves by signing up to a panel platform; or (2) they received automatic notification through a partner router which alerts/direct them to studies for which they are eligible (either via email, SMS, in-app notifications). Importantly, to avoid self-selection bias, survey invitations to eligible participants only provide general information and do not include specific details about the contents of the survey. Participants were required to be an adult (18+ years or older), able to read and write in English, and a resident of the UK. No other exclusion criteria were applied. Panel members were not obliged to take part in the study; however, panel members routinely receive an incentive for survey participation based on the length of the survey, their specific panellist profile, and target acquisition difficulty, amongst other factors. The specific type of reward varies and may include cash, air miles, gift cards, redeemable points, charitable donations, sweepstakes entrance, or vouchers. If consenting, participants accessed and completed the survey online and alone. At the time of data collection researchers has not devised the hypotheses for the current study.
Timing	The UK data collection took place between March 23rd and 28th, 2020. Data collection began 52 days after the first confirmed case of COVID-19 in the UK, and the same day the UK Prime Minister announced that people were required to stay at home except for very limited purposes. The Irish data collection took place between March 31st and April 5th, 2020. This was 31 days after the first confirmed case of COVID-19 in Ireland, 19 days after the first physical distancing measures were enacted (i.e., closure of all childcare and educational facilities), and two days after the Taoiseach (Irish Prime Minister) announced that people were not to leave their homes except for very limited purposes. Therefore, these data were collected within the first week of the strictest physical distancing

	measures being enacted in both countries.
Data exclusions	No data were excluded from analyses
Non-participation	The opt-in mode of recruitment employed by Qualtrics, albeit being a cost-effective method for gaining fast access to a large and diverse sample (and the only feasible method of recruitment during the pandemic), inevitably meant that it was not possible to generate a response rate for the baseline survey due to the lack of a known denominator or sampling frame.
Randomization	Participants were not allocated into experimental groups

Reporting for specific materials, systems and methods

We require information from authors about some types of materials, experimental systems and methods used in many studies. Here, indicate whether each material, system or method listed is relevant to your study. If you are not sure if a list item applies to your research, read the appropriate section before selecting a response.

Materials & experimental systems

n/a	Involved in the study
☒	☐ Antibodies
☒	☐ Eukaryotic cell lines
☒	☐ Palaeontology and archaeology
☒	☐ Animals and other organisms
☐	☒ Human research participants
☒	☐ Clinical data
☒	☐ Dual use research of concern

Methods

n/a	Involved in the study
☒	☐ ChIP-seq
☒	☐ Flow cytometry
☒	☐ MRI-based neuroimaging

Human research participants

Policy information about studies involving human research participants

Population characteristics	See above
Recruitment	Potential respondents were alerted to the surveys by Qualtrics in one of two ways: (1) they opted to enter studies they were eligible for by signing up to a panel platform; or (2) they received automatic notification through a partner router which alerted/directed them to studies for which they were eligible (either via email, SMS, in-app notifications). Importantly, to avoid self-selection bias, survey invitations to eligible participants only provide general information and do not include specific details about the contents of the survey.<br><br>Whilst more research is required to fully investigate the strengths and weaknesses associated with internet-based panel surveying (Bergeson, Gray, Ehrmantraut, Laibson, & Hays, 2013), it has been suggested that the composition of non-probability internet-based survey panels differs from that of the underlying population (Hays, Liu, & Kapteyn, 2015). Indeed, the American Association for Public Opinion Research (APPOR) asserts that when non-probability sampling (as opposed to probability sampling) methods are used, there is a higher burden of responsibility on investigators to describe the methods used to draw the sample and collect the data, so that users can make an informed decision about the usefulness of the resulting survey estimates (Baker et al., 2013). We support the APPOR's position that it is useful to think of different non-probability sampling approaches as falling on a continuum of expected accuracy of the survey estimates; at one end are uncontrolled convenience samples that produce risky survey estimates by assuming that respondents are a random sample of the population, whereas at the other end, there are surveys that recruit respondents based on criteria related to the survey subject matter and then the survey results are adjusted using variables that are correlated with the key study outcome variables (Baker et al., 2013). The design of our project ensures that it falls towards the latter end of the continuum.<br><br>Baker, R., Brick, J. M., Bates, N. A., Battaglia, M., Couper, M. P., Dever, J. A., . . . Tourangeau, R. (2013). Summary report of the AAPOR task force on non-probability sampling. Journal of survey statistics and methodology, 1(2), 90-143.<br><br>Bergeson, S. C., Gray, J., Ehrmantraut, L. A., Laibson, T., & Hays, R. D. (2013). Comparing web-based with mail survey administration of the Consumer Assessment of Healthcare Providers and Systems (CAHPS®) Clinician and Group Survey. Primary health care: open access, 3.<br><br>Hays, R. D., Liu, H., & Kapteyn, A. (2015). Use of Internet panels to conduct surveys. Behavior research methods, 47(3), 685-690.
Ethics oversight	Ethical approval for the study was provided by the Research Governance Committee at University of Sheffield (Reference number: 033759) and approved by the School of Psychology Ethics Filter Committee at Ulster University (Reference number: 230320)

Note that full information on the approval of the study protocol must also be provided in the manuscript.
